# Dengue Outbreak Caused by Multiple Virus Serotypes and Lineages, Colombia, 2023–2024

**DOI:** 10.3201/eid3011.241031

**Published:** 2024-11

**Authors:** Nathan D. Grubaugh, Daniela Torres-Hernández, Mónica A. Murillo-Ortiz, Diana M. Dávalos, Pio Lopez, Isabel C. Hurtado, Mallery I. Breban, Ellie Bourgikos, Verity Hill, Eduardo López-Medina

**Affiliations:** Yale University, New Haven, Connecticut, USA (N.D. Grubaugh); Rega Institute KU Leuven, Leuven, Belgium (N.D. Grubaugh); Yale School of Public Health, New Haven (N.D. Grubaugh, M.I. Breban, E. Bourgikos, V. Hill); Universidad del Valle, Cali, Colombia (D. Torres-Hernández, M.A. Murillo-Ortiz, I.C. Hurtado, E. López-Medina); Centro de Estudios en Infectología Pediátrica CEIP, Cali (D.M. Dávalos, P. Lopez, E. López-Medina); Valle del Cauca State Health Department, Cali (I.C. Hurtado); Clínica Imbanaco, Cali (E. López-Medina)

**Keywords:** Dengue, viruses, mosquitoborne diseases, vector-borne infections, outbreak, sequencing, serotypes, lineages, phylogenetics, Colombia

## Abstract

Dengue cases rose to record levels during 2023–2024. We investigated dengue in Valle del Cauca, Colombia, to determine if specific virus serotypes or lineages caused its large outbreak. We detected all 4 serotypes and multiple lineages, suggesting that factors such as climatic conditions were likely responsible for increased dengue in Colombia.

Reported cases of dengue caused by dengue virus (DENV) are increasing. DENV (genus *Orthoflavivirus*, family Flaviviridae) is composed of 4 genetically distinct serotypes, DENV-1–4. In 2023, a total of 4.6 million dengue cases were reported in the Americas, a record at the time and a 64% increase over 2022 (*1*). Those numbers were quickly surpassed in 2024, when almost 10 million dengue cases were reported through June (*1*). Of those cases, ≈8.4 million were from Brazil (*1*); however, many countries, including Colombia, reported large outbreaks. The Valle del Cauca State Health Department in Colombia reported ≈56,000 dengue cases through May 2024, compared with ≈23,000 for all of 2023 and <5,000 in 2022.

The cause of the substantial increase in dengue cases is likely multifaceted. Warming temperatures caused by climate change increase the transmission potential and expand the geographic range of the primary mosquito vector, *Aedes aegypti* (*2*). Moreover, Indian Ocean surface temperature anomalies, especially El Niño events, are associated with dengue epidemics in the Northern and Southern Hemispheres (*3*). A strong El Niño–Southern Oscillation event occurred during 2023–2024, the first since 2015–2016 (Golden Gate Weather Services, https://ggweather.com/enso/oni.htm). Moreover, new DENV introductions, perhaps related to resumption of travel after the COVID-19 pandemic (*4*), could be reaching large susceptible populations. For example, DENV-3 was rarely detected in the Americas during the 10 years before an introduction into the Caribbean from Asia around 2021 (*5*,*6*). We investigated whether a specific DENV serotype or lineage contributed to the recent surge in cases in Valle del Cauca, Colombia.

## The Study

**Figure 1:**
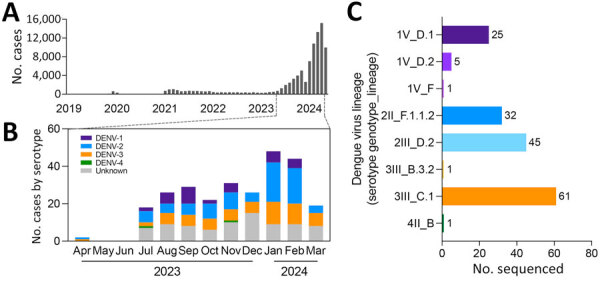
Cases in a study of multiple virus serotypes and lineages during dengue outbreak, Valle del Cauca, Colombia, 2023–2024. A) Monthly dengue cases reported by Valle del Cauca State Health Department in Colombia. Samples from confirmed dengue cases (n = 266) diagnosed at Hospital Universitario del Valle, Cali, Colombia. B) Number of cases per month by serotype during 2023–2024 period of increased dengue outbreaks. Serotypes detected by quantitative reverse transcription PCR. Samples with viral levels below detection limit are labeled unknown. C) DENV lineage by amplicon-based sequencing listed by serotype, genotype, and lineage. DENV, dengue virus.

The Valle del Cauca State Health Department in Colombia reported 966 dengue cases in 2019, 655 in 2020, 8,940 in 2021, 4,630 in 2022, 22,988 in 2023, and 56,355 cases in 2024 (through May) (Figure 1, panel A). To determine which DENV serotypes and lineages were involved, we collected 150–500 µL of serum from all 266 confirmed dengue case-patients at Hospital Universitario del Valle (HUV) in Cali, Colombia, during April 2023–May 2024. Cases were diagnosed by VIDAS anti-DENV IgM and anti-DENV IgG (bioMérieux, https://www.biomerieux.com) assays at HUV. Patient ages were 0–77 (mean 16) years, and all participants signed an informed consent; parents or guardians signed for persons <18 years of age. HUV shipped samples to Yale University (New Haven, CT, USA) for molecular processing. 

We used the QIAamp Viral RNA Mini Kit (QIAGEN, https://www.qiagen.com) to extract RNA from 140 µL of each serum sample. We initially determined DENV serotypes by using a multiplexed quantitative reverse transcription PCR (*7*) before attempting panserotype whole-genome amplicon sequencing with DengueSeq (*8*). We conducted bioinformatic analysis, including primer trimming and consensus sequence generation, by using a previously described iVar pipeline (*8*). We assigned DENV lineages to samples with >5% genome completeness, which was validated to be >93% accurate (*9*), by using the Dengue Virus Typing Tool nomenclature system (Genome Detective, https://www.genomedetective.com). We assigned serotypes to 185 (70%) samples (Figure 1, panel B); the assay was not able to detect serotypes in the remaining 81 samples because of low virus concentrations. Of the 185 samples with a serotype assignment, we assigned lineages to 171 (92%) samples via sequencing (Figure 1, panel C).

Among 2023–2024 samples, we detected all 4 DENV serotypes (DENV-1, 35; DENV-2, 85; DENV-3, 63; and DENV-4, 2) and 81 unknown serotypes (Figure 1, panel B). For part of 2023, we detected relatively equal proportions of DENV-1, DENV-2, and DENV-3, but then DENV-1 decreased as DENV-2 increased during late 2023 to early 2024. We also detected multiple lineages per serotype, except for DENV-4. DENV-3 genotype III lineage C.1 (3III_C.1 [*9*]), DENV-2III_D.2, DENV-2II_F.1.1.2, and DENV-1V_D.1 were most common (Figure 1, panel C).

To further investigate DENV lineages, we performed phylogenetic analysis using 79 sequenced samples for which we achieved >70% genome coverage: 10 DENV-1 sequences, 38 DENV-2 sequences, and 31 DENV-3 sequences. We combined our data with a background dataset downloaded from GenBank (Appendix Table) and then downsampled the data per serotype so that we kept all sequences from Colombia, 5 per year from the other countries in the Americas, and 1 per year from each country outside the Americas (1,007 DENV-1, 965 DENV-2, and 542 DENV-3 sequences). We analyzed the sequences using the Nextstrain bioinformatic and phylogenetic framework (*10*), and constructed maximum-likelihood trees using IQ-TREE (*11*) and a general time-reversible substitution model. 

**Figure 2:**
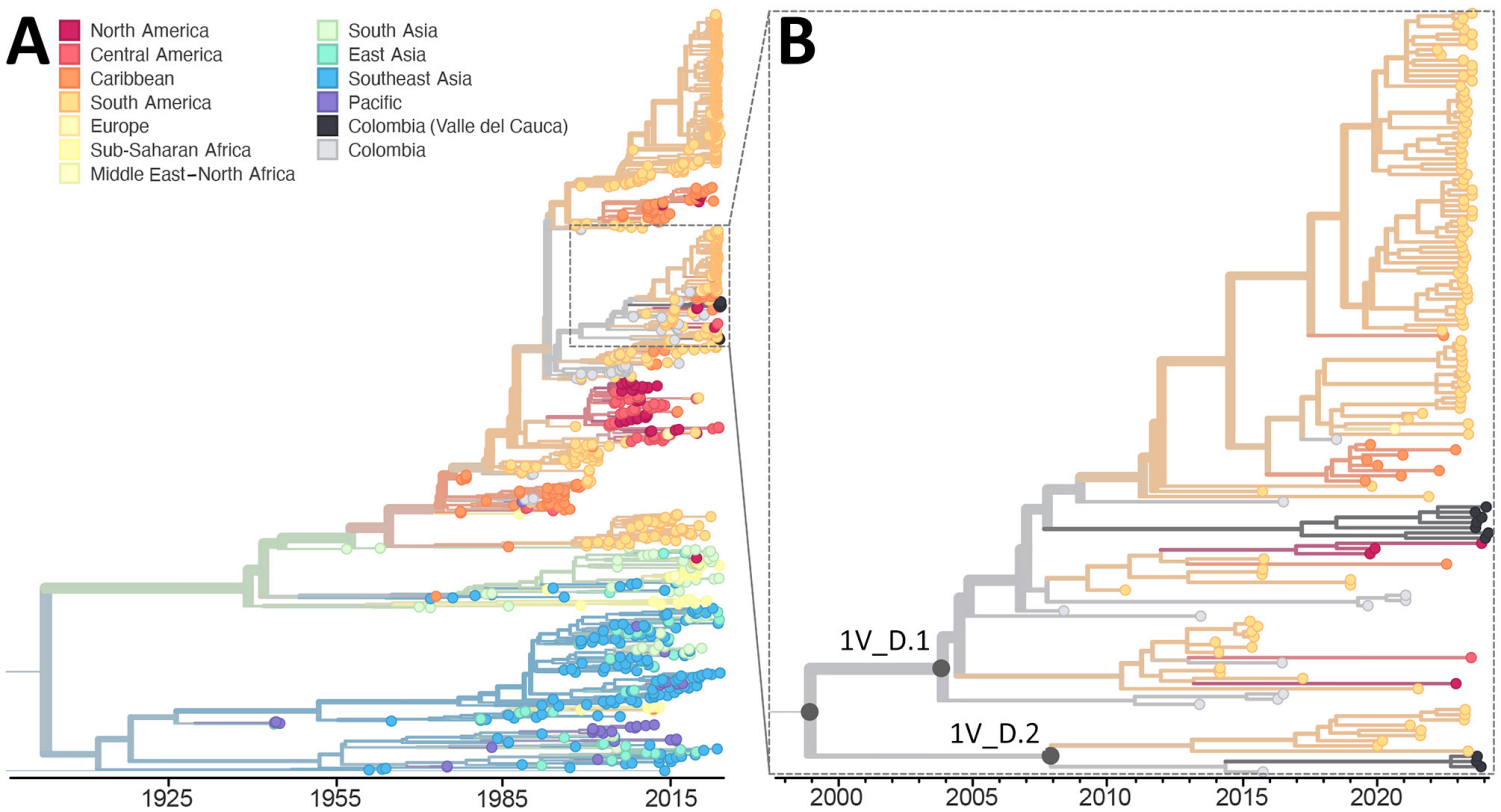
Time-resolved maximum-likelihood phylogeny of DENV-1 detected during an investigation of multiple virus serotypes and lineages during dengue outbreak, Valle del Cauca, Colombia, 2023–2024. The tree includes global DENV-1 sequences downloaded from GenBank and was constructed by using IQ-TREE (http://www.iqtree.org). A) Full reconstruction of 1,007 DENV-1 sequences from 1944–2024 colored by sampling location. B) Detail of the DENV-1V_D clade highlighting sequences from Valle del Cauca, Colombia (black) from 2023 through mid-2024. DENV, dengue virus.

Our DENV-1 phylogenetic analysis revealed cocirculation of 2 distinct lineages, DENV-1V_D.1 and D.2 (Figure 2). Both lineages were previously detected in Colombia and elsewhere in South America (*5*,*12*), representing ongoing local and regional lineage persistence and diversification for the past ≈15–20 years.

**Figure 3:**
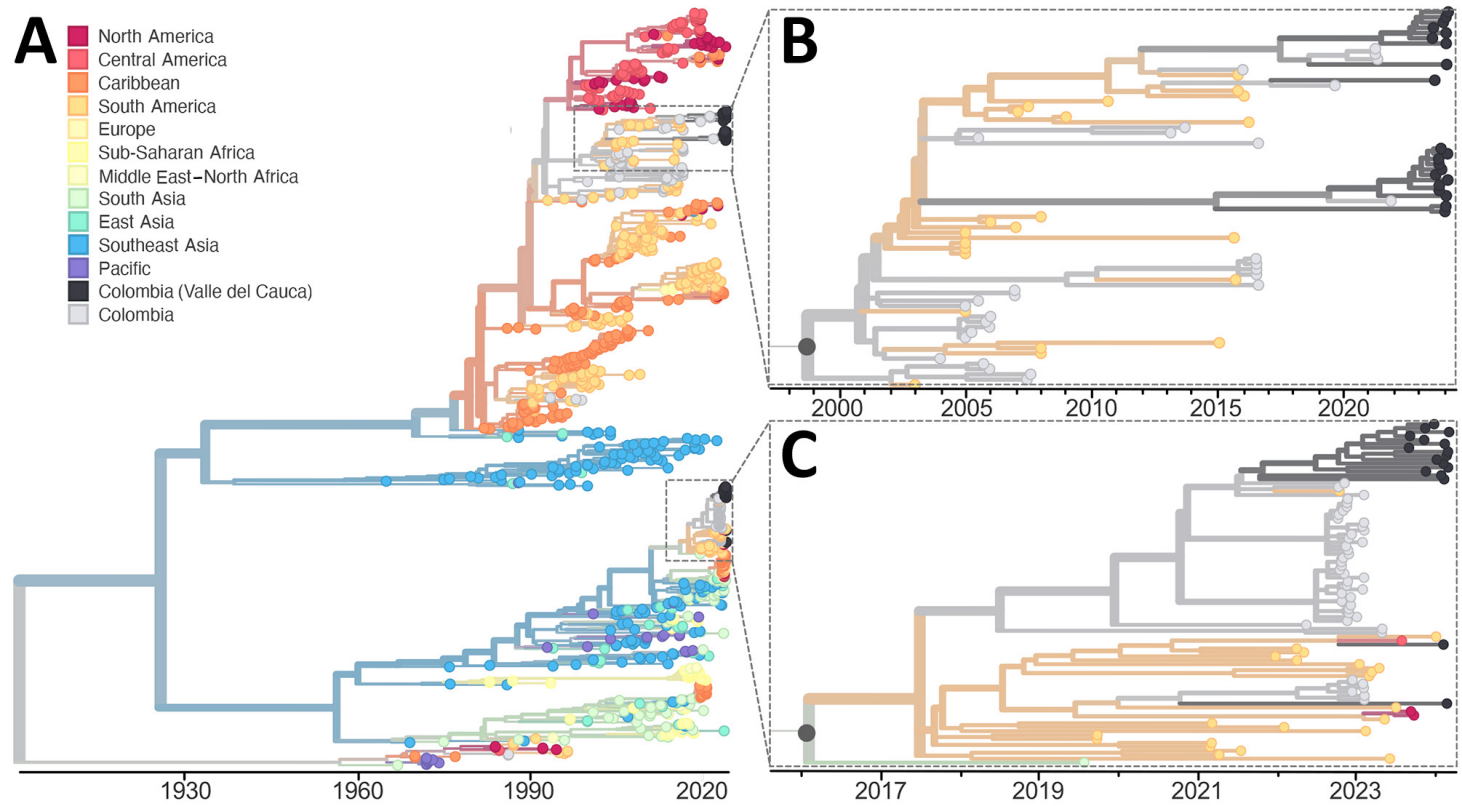
Time-resolved maximum-likelihood phylogeny of DENV-2 detected during an investigation of multiple virus serotypes and lineages during dengue outbreak, Valle del Cauca, Colombia, 2023–2024. The tree includes global DENV-2 sequences downloaded from GenBank and was constructed by using IQ-TREE (http://www.iqtree.org). A) Full reconstruction of 965 DENV-2 sequences from 1964­–2024 colored by sampling location. B) Detail of the DENV-2III_D.2 clade highlighting sequences from Valle del Cauca, Colombia (black) from 2023 through mid-2024. C) Detail of DENV-2II_F.1.1.2 clades highlighting sequences from Valle del Cauca, Colombia (black) from 2023 through mid-2024. DENV, dengue virus.

Our DENV-2 phylogenetic analysis presents a more complicated picture of 3 genetic clusters and 3 individual sequences dispersed among 2 defined lineages, DENV-2III_D.2 and DENV-2II_F.1.1.2 (Figure 3). Lineage 2III_D.2 is a descendent of the original DENV-2 genotype III (i.e., Asian-American lineage) that was introduced in the Americas during the late 1970s and subsequently became established throughout the region, including in Colombia (*13*). DENV-2 genotype II (a.k.a. Cosmopolitan lineage) was recently introduced into the Americas from Asia and was first detected during a dengue outbreak in Peru in 2019 (*14*). Detection of DENV-2II_F.1.1.2 in Valle del Cauca demonstrates that the emerging Cosmopolitan genotype can become established alongside the existing Asian-American genotype.

**Figure 4:**
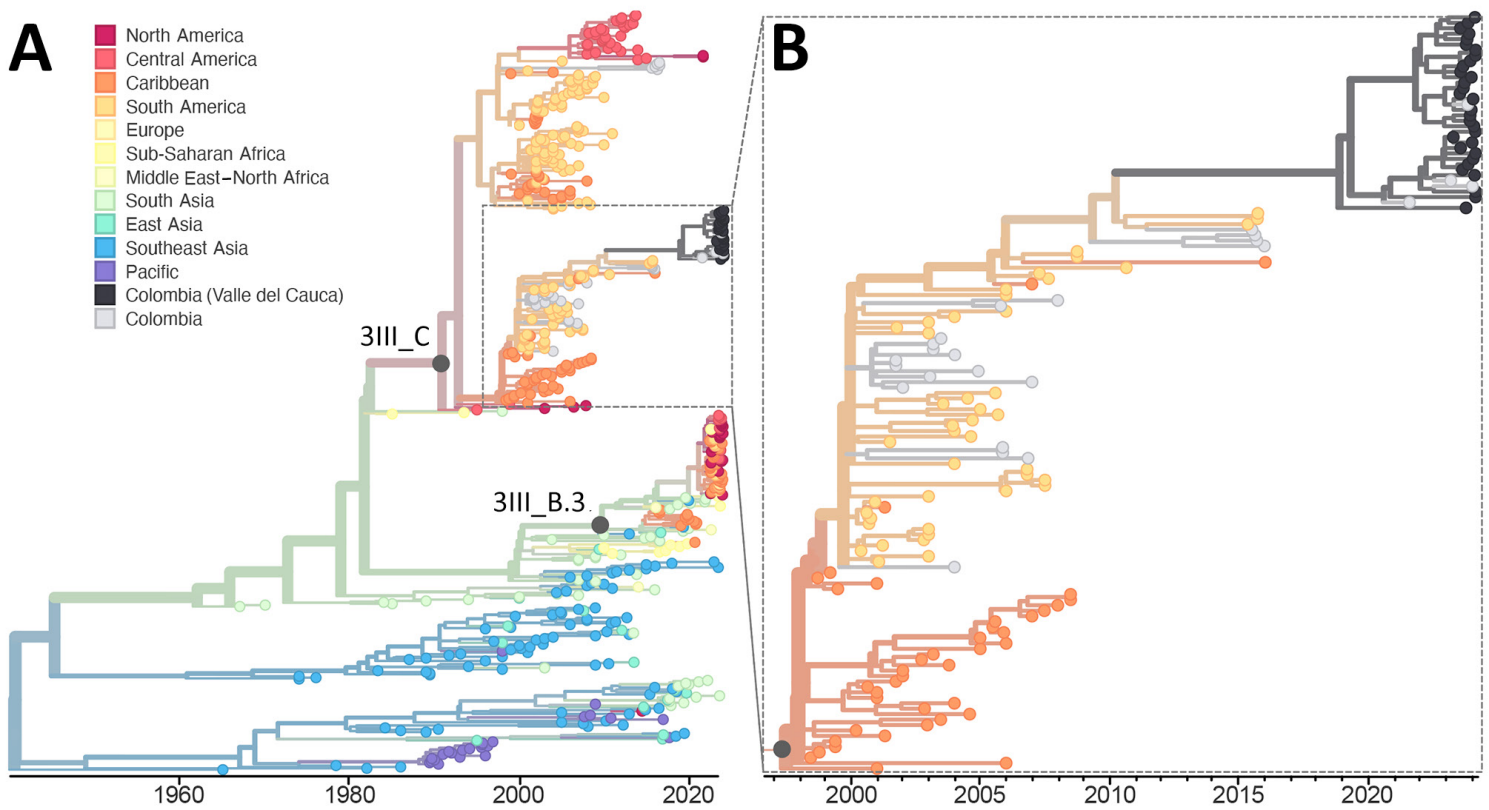
Time-resolved maximum-likelihood phylogeny of DENV-3 detected in an investigation of multiple virus serotypes and lineages during dengue outbreak, Valle del Cauca, Colombia, 2023–2024. The tree includes global DENV-3 sequences downloaded from GenBank and was constructed by using IQ-TREE (http://www.iqtree.org). A) Full reconstruction of 542 DENV-3 sequences from 1964 through 2024 colored by sampling location. B) Detail of the DENV-3III_C.1 clade highlighting sequences from Valle del Cauca, Colombia (black) from 2023 through mid-2024.

One hypothesis for the sudden increase in dengue cases is the introduction and rapid spread of a new DENV-3 lineage from Asia (*5*). DENV-3 can go undetected for long time periods in the Americas, sometimes for more than a decade, leaving large portions of the population potentially susceptible to this serotype (*4*,*6*,*15*). Therefore, detection of an emerging DENV-3III_B.3.2 lineage in the Caribbean (*5*), Brazil (*6*), Nicaragua (*4*), and elsewhere in the Americas was alarming. We detected 1 dengue case from January 2024 in Valle del Cauca with a likely 3III_B.3.2 infection (18% genome coverage), but 97% (61/63) of DENV-3 infections were lineage 3III_C.1 (Figure 1, panel C), and the lineage from 1 DENV-3 infection could not be assigned. DENV-3III_C was likely first introduced into the Americas in the early 1990s (*13*). Our findings show that DENV-3III_C has persisted through long periods of low detection (Figure 4), including sporadic detections of 3III_C.1 in Colombia since the early 2000s. Therefore, our results suggest that populations in the Americas might be susceptible to DENV-3 in general and not just the emerging 3III_B.3.2 lineage.

## Conclusions

We investigated DENV infections from Valle del Cauca, Colombia, to determine if a specific virus serotype or lineage might be driving the record number of dengue cases in that state (*1*). We detected all 4 serotypes and found DENV-1, DENV-2, and DENV-3 shared dominance and at least 8 separate defined lineages were involved. Those lineages included multiple DENV-1 genotype V and DENV-2 genotype III lineages that have circulated in the Americas for ≈40 years (*13*), as well as an emerging DENV-2 genotype lineage. Moreover, despite the rapid spread of a new DENV-3III_B.3.2 lineage in the Americas (*4*–*6*), we found that the dominant DENV-3 lineage was 3III_C.1, which has been sporadically detected in Colombia for ≈20 years. Although multiple DENV serotypes are often detected during endemic transmission, our results were unexpected because outbreaks are typically dominated by a single serotype. 

In summary, DENV lineages can have variable phenotypes that affect virulence, transmissibility, and immune evasion. Detecting several co-dominating serotypes and lineages in Valle del Cauca suggests that the specific viruses were not the primary driver of the large outbreak. Our study demonstrates how genomic surveillance can help investigate causes of outbreaks and aid public health responses.

Appendix Genomic data on multiple virus serotypes and lineages during dengue outbreak, Valle del Cauca, Colombia, 2023–2024.
